# Ribosome purification from *Escherichia coli* by ultracentrifugation

**DOI:** 10.1016/j.biotno.2022.12.003

**Published:** 2022-12-08

**Authors:** Yi Cui, Xinjie Chen, Ze Wang, Yuan Lu

**Affiliations:** a College of Life Sciences, Shenyang Normal University, Shenyang, 110034, Liaoning, China; b Key Laboratory of Industrial Biocatalysis, Ministry of Education, Department of Chemical Engineering, Tsinghua University, Beijing, 100084, China

**Keywords:** *Escherichia coli*, Ribosome extraction, Ultracentrifugation

## Abstract

With more and more researchers conducting extensive research on all aspects of ribosomes, how to extract ribosomes with good effect and high activity has become a fundamental problem. In this article, *Escherichia coli* A19, MRE600, and JE28 cells often mentioned in the literature and ordinary *E. coli* BL21(DE3) cells were used to extract ribosomes by ultracentrifugation. The purpose was to study whether the ultracentrifugation method can be applied to extract effective ribosomes, and whether the ribosome extracts from different cells were different. The extracted ribosomes were validated by RNA electrophoresis, SDS-PAGE, PURE system, and mass spectrometry. The validation experiment results showed that ribosomes from these four cells had different effects. The success of the experiment confirmed that effective ribosomes could be extracted from *E. coli* by ultracentrifugation, which laid a good foundation for researchers to carry out further applications on ribosomes.

## 1 Introduction

The ribosome is a highly complex cellular machine in cells, mainly composed of 35% r-protein and 65% rRNA.^1^^,^^2^ Its function is to convert the genetic code into amino acid sequences according to the instructions of mRNA and construct protein polymers from amino acid monomers. Therefore, ribosomes are also called molecular machines for protein synthesis in cells. As the most significant of organelles in the cell,^3^ researchers have concerned ribosome in all aspects.

The research on ribosomes mainly focuses on four aspects. First, the early research is to understand the composition and structure of ribosomes. Second, the mechanism of ribosome biosynthesis is further explored. For example, the abnormality of ribosome biosynthesis may promote the occurrence and development of tumors, which may become a feasible therapeutic target for multiple tumors.^4^ Third, the role of proteins in ribosomes is investigated. For example, some researchers found that gene knockout ribosomal BS22 and BL37 proteins could enhance the sensitivity of mycobacteria to antibiotics, suggesting that ribosomal proteins could be used as drug design targets.^5^ Fourth, biological functions of ribosome in health and other applications are developed. For example, in a blockbuster article published by Nature this year, it is stated that increased ribosome pausing would lead to ribosome-associated quality control overload and aggregation of nascent polypeptides, thus accelerating the imbalance of protein stability and systematic decline during aging.^6^ The above research on various aspects of ribosomes can tell the importance of ribosomes. Their acquirement is the first step to performing all aspects of research. However, the first thing to bear the brunt is that ribosomes extracted from cells are often affected by the environment, resulting in RNA degradation and protease pollution, which affect the activity of ribosomes. How to purify ribosomes with good activity is a problem that researchers are still constantly exploring.

At present, there are two main purification methods for ribosomes. One is introducing His-tag into ribosomal subunits in advance, and the ribosome can be purified by Ni-NTA Sepharose. Experiments have proved that such ribosome purification methods can purify active ribosomes, but their activity is lower than that of unlabeled variants.^7^^,^^8^ There is a difficulty in the purification process of ribosomes in this way; that is, ribosomes are easy to degrade. The other is the traditional purification method, namely, ultracentrifugation. It removes most of the cell fragments through cell lysis and centrifugation in the early stage, and then obtains ribosome precipitation through ultracentrifugation.

Ultracentrifugation is a method of separating, concentrating, and purifying substances according to the sedimentation coefficient, mass, density and other factors of different substances by using huge centrifugal force. During centrifugation, each component sinks at a different speed, but eventually stays in the same density as itself, forming a narrow equilibrium zone and maintaining relative stability. Moreover, ultracentrifugation can be operated at low temperature, which is not only more widely used in all cells, but also conducive to protecting the activity of ribosomal macromolecules. Generally, centrifugation is required for a long time in order to make all components of the sample reach their equilibrium position.^9^^,^^10^ However, in this protocol, by adding sucrose solution during ultracentrifugation, the sucrose solution can quickly form a gradient, which greatly shortens the ultracentrifugation time to 1–2h, and still maintains a good separation effect. The sucrose solution combined with ultracentrifugation can be a convenient and quick method.

In principle, ribosomes can be extracted from any cell by ultracentrifugation. *Escherichia coli* strains A19, MRE600, JE28, and BL21 (DE3) were selected in this study. MRE600 lacks RNase I, and it is a commonly used preparation bacterium for separating ribosomes by chromatography.^11^, ^12^, ^13^ A19 also lacks RNase I, and it has six mutations (As shown in Table 1), which were used by researchers to successfully extract ribosomes through the FPLC system and sucrose density gradient centrifugation.^10^^,^^14^^,^^15^ JE28 has His-tag, and researchers have used HisTrap HP column to extract ribosomes.^16^ The researchers have successfully obtained ribosomes from these three strains, but they do not simply use ultracentrifugation. In order to verify the universality of ultracentrifugation, *E. coli* BL21(DE3), which is very common in the laboratory, was added in this study. The ribosome was extracted by providing the same culture conditions and ultracentrifugation. RNA electrophoresis, SDS-PAGE, protein synthesis using recombinant elements (PURE) system, and mass spectrometry analysis further verified the ribosome quality and activity from different cells.

**Table 1:** Selected cells and their respective characteristics.

Strain	Characteristic	Reference
A19 (CGSC 5997)	6 mutations: rna-19, gdhA2, his-95, relA1, spoT1, metB1.	^17^
MRE600 (ATCC 29417)	Lacks Rnase I	^11^
JE28	In-frame fusion of a nucleotide sequence encoding a hexa-histidine affinity tag at the 3′-end of the single copy rplL gene (encoding the ribosomal protein L12) at the chromosomal site of the wild-type strain MG1655.	^16^
BL21(DE3)	No special treatment, selected as control.	Commercial

## 2 Experimental designs

**Fig. 1:**
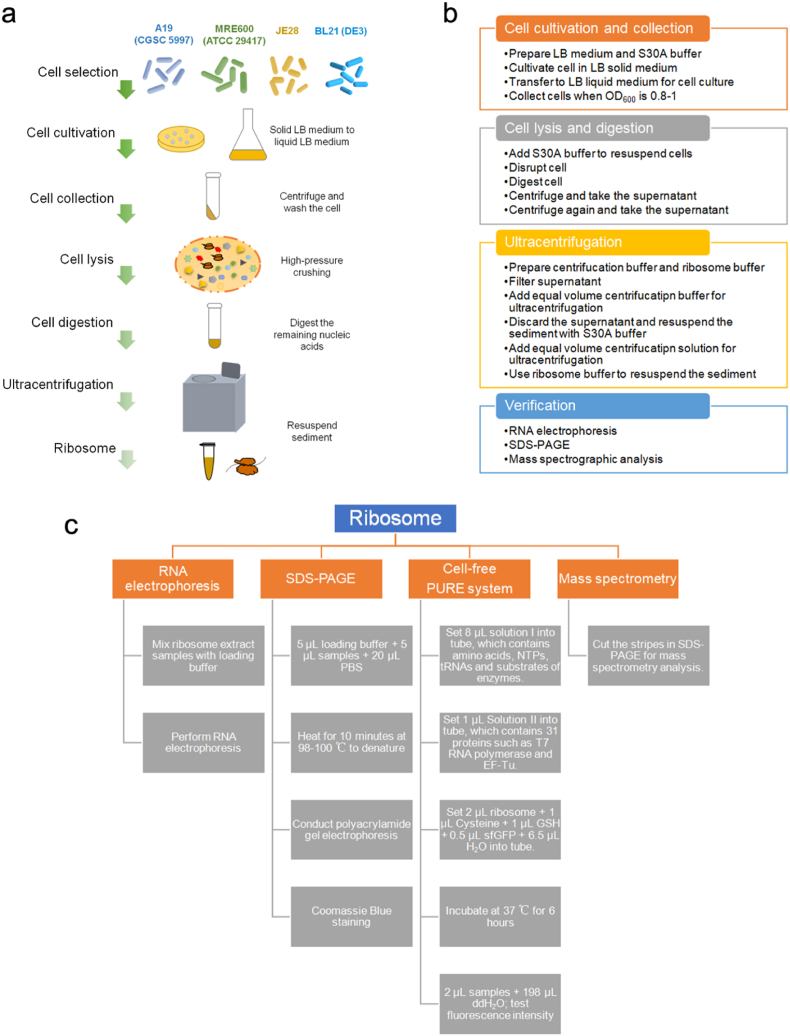
The process of extracting ribosome from *E. coli* cells by ultracentrifugation (a), the experimental flow chart of this protocol (b), and the main flow chart of ribosome activity and physical property analysis (c).

This protocol describes how to obtain ribosome extract from *E. coli* by ultracentrifugation. Based on previous literature research, this method selected *E. coli* strain cells, including A19 (CGSC 5997),^17^ MRE600 (ATCC 29417),^11^ and JE28^16^ used by predecessors to extract ribosomes, as well as commonly used *E. coli* cell BL21(DE3). The characteristics of each cell are shown in Table 1. The experiment procedure is mainly divided into four steps, as shown in Fig. 1a. First, cultivate and collect cells. The culture medium was slowly cooled from 37 °C to 4 °C to produce run-off ribosomes.^16^ Second, perform fragmentation and digestion. The purpose of digestion is to digest the remaining nucleic acids by using the released endogenous exonucleases. Third, obtain ribosome precipitation by ultracentrifugation. Fourth, the activity and physical characterization of ribosomes were verified.

### 2.1 Materials

#### 2.1.1 Preparation of cell culture and collection

• A19 (CGSC 5997)
• MRE600 (ATCC 29417)
• JE28
• BL21(DE3)
• Yeast Extract (OXIOD; Cat. no.: LP0021)
• Tryptone (OXIOD, Basingstoke, UK; Cat. no.: LP0042)
• Nacl (Sinopharm Chemical Reagent, Shanghai, China; Cat. no.: 10019318)
• PBS buffer (Solarbio; Cat. no.: P1010)

#### 2.1.2 Cell lysis and digestion

• Potassium L-glutamate (Yuanye, Shanghai, China; Cat. no.: S20427)
• L-Glutamic acid hemimagnesium salt tetrahydrate (Sigma-Aldrich; Cat. no.: 49605)
• Tris (Biotopped, Beijing, China; Cat. no.: T6061)

#### 2.1.3 Ultracentrifugation

• Tris (Biotopped, Beijing, China; Cat. no.: T6061)
• Ethylenediaminetetraacetic acid (EDTA) (Solarbio; Cat. no.: E8040)
• β-mercaptoethanol (Solarbio; Cat. No.:M8210)
• 2-[4-(2-hydroxyethyl)piperazin-1-yl]ethanesulfonic acid (HEPES) (Solarbio; Cat. no.: H8090)
• Potassium chloride (Sinopharm Chemical Reagent, Shanghai, China; Cat. no.: 10016318)
• Magnesium acetate (Macklin, Shanghai, China; Cat. no.: M833330)
• Ammonium chloride (Sinopharm Chemical Reagent, Shanghai, China; Cat. no.: 10001518)
• Sucrose (Sinopharm Chemical Reagent, Shanghai, China; Cat. no.: 10021418)

### 2.2 Equipment

#### 2.2.1 Preparation of bacterial culture

• 1 L flasks
• Constant temperature shaker
• Ultrospec 3100 pro UV/Visible spectrophotometer (Amersham, Piscataway, NJ, USA)
• Micro Refrigerated Centrifuge Model 3700 (KUBOTA, Osaka, Japan)
• MS-H-Pro+ magnetic stirring apparatus (DRAGONLAB, Beijing, China)
• Vortex-Genie 2 (Scientific Industries)

#### 2.2.2 Cell lysis and digestion

• JN-3000PLUS high press crusher (JNBIO, China)
• Vortex-Genie 2 (Scientific Industries)
• Constant temperature shaker
• Micro Refrigerated Centrifuge Model 3700 (KUBOTA, Osaka, Japan)

#### 2.2.3 Ultracentrifugation

• Ultracentrifuge
• Ultracentrifuge Rotor SW41 Ti
• Ultra-clear centrifuge tubes (BECKMAN COULTER, cat. no. 344059)
• Millex® 33mm PES .22 um

#### 2.2.4 The activity and physical characterization of ribosomes were verified

• Infinite M200 PRO Microplate Reader (Tecan, Switzerland)
• Mini-PROTEAN® Tetra Cell (Bio-rad)

## 3 Procedure

The experiment is divided into four parts, as shown in Fig. 1b, including cell cultivation and collection, cell lysis and digestion, ultracentrifugation, and characterization analysis.

### 3.1 Cell culture and collection (time for completion: 3–4 days)

1. Prepared solutions used in this part
  • LB medium: 5 g/L Yeast extract, 10 g/L Tryptone, 10 g/L NaCl (and 1.5% agar for plate).
  • S30A buffer: 14 mM L-Glutamic acid hemimagnesium salt tetrahydrate, 60 mM Potassium L-glutamate, 50 mM Tris, pH 7.7, titrated with acetic acid.
2. Prepared and autoclaved LB medium (10 mL, 200 mL, and 4 L in the bioreactor).
3. Cultivated strain on LB solid medium, and incubated overnight at 37 °C.
4. Selected single colony and transferred it to 10 mL liquid LB medium in 50 mL flask and incubated overnight at 37 °C with 220 rpm.
5. Transferred 10 mL overnight culture into 200 mL fresh LB medium in 1 L flask and continued culturing for about 2 h in the shaker.

**CRITICAL:** In order to make the bacteria have better activity after multistage culture, when the secondary bacteria grew to the logarithmic growth period, they were transferred to the final medium for cultivation.

6. Inoculated the cultured bacteria into 4 L LB medium prepared in advance according to 5% of the inoculation amount, and stopped the cultivation when the OD_600_ was 0.8–1 at 37 °C and 220 rpm.
7. Centrifuged at 4000 rpm for 30 min at 4 °C to collect bacteria.

**CRITICAL**: The culture medium was slowly cooled from 37 °C to 4 °C to produce run-off ribosomes.

8. Used 1 × PBS buffer to resuspend the cell, centrifugated it at 10000 rpm for 10 min at 4 °C, washed the cell three times, and weighed the cell weight.

**CRITICAL:** Used 1 × PBS buffer to wash the residual medium liquid as much as possible. Before weighing cells, removed the residual PBS buffer as much as possible.

**PAUSE POINT:** After being washed, the pellet could be stored at 4 °C overnight or at −80 °C for a long time.

### 3.2 Cell lysis and digestion (time for completion: 1 day)

1. Added the S30A buffer prepared in advance to the tube at the rate of 1 mL/g, and resuspended the cells completely by stirring.
2. Subjected the suspension to a high-press crusher (JNBIO) twice at 15000–20000 psi.
3. The tube containing the broken cells was placed at 37 °C and 220 rpm for 80-min digestion.
4. Centrifuged the lysed cells at 4 °C and 10000 rpm for 60 min.

**CRITICAL:** Removed unnecessary cell fragments.

5. Transferred the supernatant into a clean centrifuge tube and centrifuged the lysed cells at 4 °C and 10000 rpm for 60 min again.

**CRITICAL**: This step should be carried out on a clean bench.

### 3.3 Ultracentrifugation (time for completion: 1 day)

1. Prepared solutions used in this part
  • Ultracentrifugation buffer: 20 mM Tris (pH 7.7, titrated with hydrochloric acid), 500 mM ammonium chloride, 10.5 mM magnesium acetate, 0.5 mM EDTA, 7 mM β-mercaptoethanol, and 30% sucrose.
  • Ribosome buffer: 20 mM HEPEs (pH 7.7, titrated with potassium hydroxide), 6 mM magnesium acetate, 30 mM potassium chloride, and 7 mM β-mercaptoethanol.
2. The newly obtained supernatant was filtered through a sterile filter membrane (0.22 μm).
3. Filled the obtained sample and centrifugation buffer into the ultracentrifugation tube in a volume of 1:1.
4. Ultracentrifuged at 4 °C by 170000 g for 2 h.
5. Discarded the supernatant, used S30A buffer to resuspend the sediment. Filled the S30A buffer and Ultracentrifugation buffer into the ultracentrifugation tube in a volume of 1:1. Ultracentrifuged at 4 °C by 170000 g for 2 h again.

**CRITICAL:** The sediment was resuspended on ice, and S30A buffer was precooled to low temperature in advance.

6. Discarded the supernatant, used 500 μL Ribosome buffer to resuspend the sediment, and transferred it to a clean 1.5-mL sterile centrifuge tube.

**CRITICAL:** The sediment was resuspended on ice, and Ribosome buffer was precooled to low temperature in advance.

**PAUSE POINT**: At this time, the samples could be repackaged and frozen for use in subsequent test experiments.

### 3.4 Verifying the activity and physical characterization of ribosomes (time for completion: 2–3 days)

#### 3.4.1 Quantitative characterization of purified ribosomes

1. Analyzed the samples by RNA electrophoresis.
2. 5 μL samples from each cell ribosome extract were diluted with 20 μL PBS.
3. Added 5 μL 6 × protein loading buffer for sodium dodecyl sulfate polyacrylamide gel electrophoresis (SDS-PAGE).

#### 3.4.2 Validation experiment of PURE system

1. Thaw PURE components and ribosome samples on ice.
2. Used the superfolder green fluorescent protein (sfGFP) plasmid as the template for the experiment, and the standard 20 μL Reaction mixture of PURE system included: 8 μL Solution 1, 1 μL Solution 2, 2 μL Ribosome, 1 μL GSH, 1 μL Cysteine, 0.5 μL sfGFP plasmid, and 6.5 μL H_2_O.
3. Mixed the mixture and reacted at 37 °C for 6 h.
4. 2 μL samples from each reaction mixture were diluted with 198 μL ddH_2_O, and the fluorescence intensity of these diluted samples was measured with F485 excitation and F535 emission filters using Microplate Reader.

#### 3.4.3 Mass spectrometry analysis

Analysis of samples was performed at the Center of Biomedical Analysis of Tsinghua University.

## 4 Results

In this experiment, each cell was cultivated with 4-L volume. At the end of ultracentrifugation, added 500 μL ribosome buffer to the precipitate to obtain resuspended ribosome extract. The obtained ribosome extract was tested on ribosome activity and physical characteristics. The experiments were mainly divided into four parts, including RNA electrophoresis, SDS-PAGE, PURE system verification, and mass spectrometry analysis, and the main process of each part was shown in Fig. 1c.

### 4.1 Quantitative characterization of purified ribosomes

The ribosome is mainly composed of RNA and protein, so RNA electrophoresis is used to examine RNA in the ribosome, and SDS-PAGE is used to examine protein in the ribosome.

**Fig. 2:**
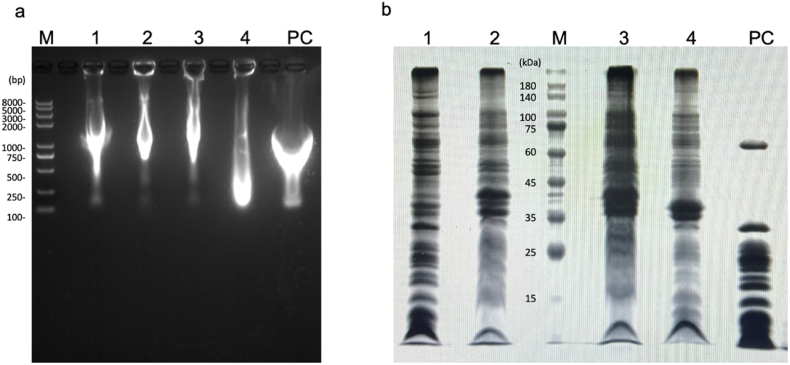
RNA electrophoresis and SDS-PAGE of ribosome extracts and commercial ribosomes. M: Marker; 1: Ribosome extracts from A19 cells; 2: Ribosome extracts from MRE600 cells; 3: Ribosome extracts from JE28 cells; 4: Ribosome extracts from BL21(DE3) cells; PC: Positive control made by commercial ribosomes.

RNA electrophoresis was carried out on ribosome extracts from A19, MRE600, JE28 and BL21(DE3) cells. In order to know which cell was better, commercial ribosomes were used for comparison. As shown in Fig. 2a. The RNA range of commercial ribosomes was 100–2000 bp, most of which are concentrated in 300–1800 bp. The RNA range of ribosome extracts from A19 cells was 400–3000 bp, mainly concentrated in 500–2000 bp. The RNA range of ribosome extracts from MRE600 and JE28 cells was 750–8000 bp, mainly concentrated in 750–2000 bp. The RNA of the ribosome extract of BL21 (DE3) cells was presented in 100–8000 bp, but mainly concentrated in 100–500 bp. From the position and shape of RNA, the ribosome extract of A19 cell was the most similar to the results of commercial ribosomal RNA, followed by MRE600 cell ribosome extract and JE28 cell ribosome extract, and BL21(DE3) cell ribosome extract is the most dissimilar. The reason for this result might be that BL21 (DE3) had not been specially modified, and the RNA contained in BL21(DE3) was largely degraded during the extraction process. The A19, MRE600 and JE28 cells with different modifications had different anti-RNA degradation capabilities. Therefore, when selecting the cells for ribosome extraction, it was better to select cells with modifications to RNases.

In addition to RNA electrophoresis, SDS-PAGE was conducted to verify the results, as shown in Fig. 2b. From the SDS-PAGE results, the protein bands contained in commercial ribosomes were found in the ribosome extracts of A19, MRE600, JE28 and BL21 (DE3). However, it could be seen that the ribosome extracts obtained from four kinds of *E. coli* cells contained a large number of foreign mixed proteins. If there were any carelessness during the experiment, there would be mixed proteins involved, which could affect the ribosome functions.

### 4.2 Using PURE system to verify the ribosome function

**Fig. 3:**
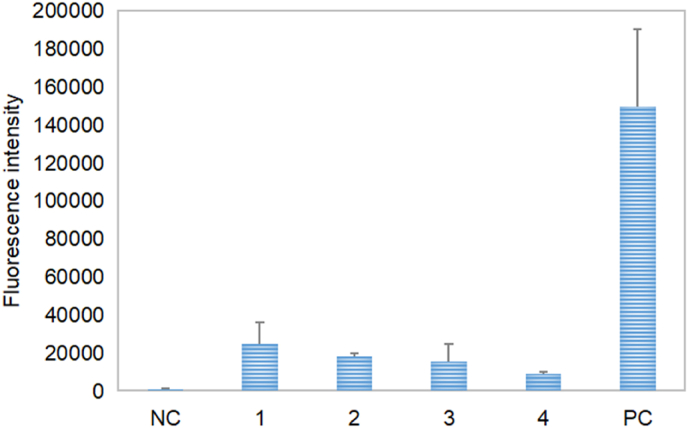
Cell-free expression results of PURE system constructed by ribosomes extracted from different cells. NC: Negative control without sfGFP template; 1: Ribosome extracts from A19 cells; 2: Ribosome extracts from MRE600 cells; 3: Ribosome extracts from JE28 cells; 4: Ribosome extracts from BL21(DE3) cells; PC: Positive control made by commercial ribosomes.

As the core of the translation mechanism, ribosome is the most critical component of cell-free PURE system. The ribosome extracts directly affected the success and failure of cell-free PURE system. sfGFP expression plasmid was used as the DNA template. The ribosome extracts from four different cells were applied to cell-free PURE system. As shown in Fig. 3, cell-free expression results showed that although there was a gap between the results of PURE system and those of commercial ribosomes, four experimental groups had successfully built PURE system. In four experimental groups, the ribosome extract of A19 cells was still the best, followed by MRE600 and JE28, and the ribosome extract from BL21 (DE3) cells was still the worst. Through T-test analysis on PURE system results of four experimental groups, it was found that although the results of ribosomes extracted by MRE600 and JE28 for PURE system were lower than those of ribosomes extracted by A19 for PURE system, they were not enough to form significant differences. After comparing the four experimental groups, only the ribosomes extracted from A19 and BL21 (DE3) were significantly different in PURE system. There was a gap between the results of PURE system established by ribosome extract and commercial ribosome respectively. According to SDS-PAGE results, there were a lot of foreign mixed proteins in ribosome extract. Its existence affected the concentration and quality of ribosomes. In the future, the existence of foreign mixed proteins needs to be further improved through continuous research. Only by comparing the experimental group, A19 and MRE600 ribosomes had a good effect, which was largely due to the positive impact of gene mutation and RNase I knockout. The poor effect of JE28 ribosome was probably due to His-tag's influence on it. BL21 (DE3), without any modification, was faced with problems of both RNA degradation and foreign protein pollution. The cell-free reaction results of the PURE system were consistent with the result of RNA electrophoresis above.

### 4.3 Validation of ribosome proteins by mass spectrographic analysis

**Fig. 4:**
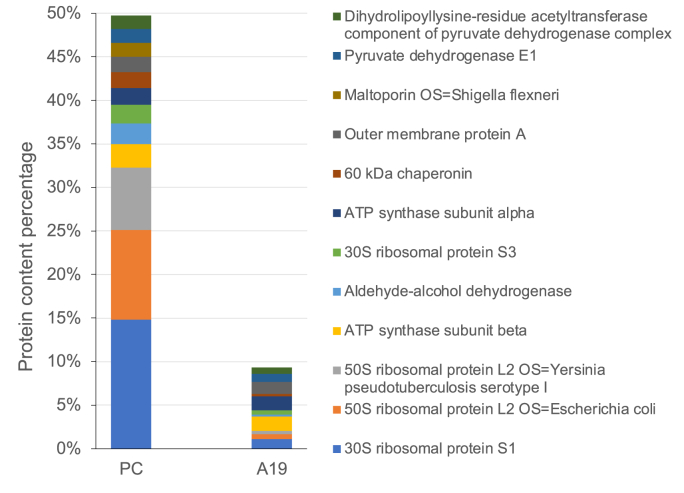
Typical proteins in the ribosome extract of A19 cells and their comparison with commercial ribosomes. PC: Positive control made by commercial ribosomes.

The ribosome of *E. coli* is mainly composed of large subunits and small subunits. There are about 22 proteins (numbered S1 to S22) in the small subunit, and about 34 proteins (numbered L1 to L36) in the large subunit of the ribosome.^18^ In order to further understand the composition of proteins in ribosome extracts, A19 ribosomes with the best results and commercial ribosomes were analyzed by mass spectrometry. By arranging the proteins in commercial ribosomes from high to low, the first 12 proteins accounting for about 50% of the total protein, were shown in Fig. 4. Among the 12 proteins, only one was not detected in the ribosome extract of A19 cells, and it was maltoporin. The remaining 11 proteins were detected, and their total amount accounted for less than 10% of the total protein content of the A19 ribosome extract. The results of mass spectrometry analysis and SDS-PAGE confirmed the previous conjecture. The A19 ribosome extract contained a large number of foreign proteins in the ribosome. How reduce the contamination of foreign proteins would become one key research direction of ribosome purification in the future.

## 5 Conclusion

Ribosomes have been widely concerned and constantly explored by researchers since they were discovered in the early 1950s. While researchers dig deeply into its various aspects, they also increasingly hope to extract effective and active ribosomes. Although there have been some different methods to extract ribosomes, the most widely used method is still ultracentrifugation. In this study, ultracentrifugation can not only obtain ribosomes quickly, conveniently, and effectively, but also it is not limited by the cells used. Although different kinds of *E. coli* cells can all be used to obtain ribosomes by ultracentrifugation, it is better to select cells with modifications to RNases, such as A19 cells. A series of validation experiments were carried out, such as RNA electrophoresis, SDS-PAGE, cell-free reaction, and mass spectrometry analysis, to confirm the quality of ribosomes extracted by ultracentrifugation. Although effective ribosome extracts were obtained by ultracentrifugation, the validation results also showed that there were many uncertain foreign proteins in the extracted ribosomes, leading to relatively low ribosome activity. In future studies, more convenient approaches need to be developed to get high-quality ribosomes, and more effective mutations could be made to prevent RNA degradation and foreign protein contamination.

## Declaration of competing interest

The authors declare that they have no known competing financial interests or personal relationships that could have appeared to influence the work reported in this paper.

## References

[1] K C. (1960). Molecular characterization of ribonucleic acid from Escherichia coli ribosomes : I. Isolation and molecular weights. J Mol Biol.

[2] Wilson D.N., Doudna Cate J.H. (2012). The structure and function of the eukaryotic ribosome. Cold Spring Harbor Perspect Biol.

[3] Li-Yu Z., Ren-Qi Y., Yong-Ming Y. (2022). Update Advances in Ribosome-associated Quality Control and Ribophagy. Prog Biochem Biophys.

[4] Qian Lili, Honghe Z. (2020). Research Progress of ribosome Biogenesis and cancer. Cancer Res Prevent Treat.

[5] Shan C., Yue Q., Ding X. (2022). [Knockout of ribosomal genes bS22 and bL37 increases the sensitivity of mycobacteria to antibiotics]. Sheng Wu Gong Cheng Xue Bao.

[6] Stein K.C., Morales-Polanco F., van der Lienden J. (2022). Ageing exacerbates ribosome pausing to disrupt cotranslational proteostasis. Nature.

[7] Wang H.H., Huang P.Y., Xu G. (2012). Multiplexed in vivo His-tagging of enzyme pathways for in vitro single-pot multienzyme catalysis. ACS Synth Biol.

[8] Grasemann L., Lavickova B., Elizondo-Cantú M.C. (2021). OnePot PURE cell-free system. JoVE.

[9] Rivera M.C., Maguire B., Lake J.A. (2015). Isolation of ribosomes and polysomes. Cold Spring Harb Protoc.

[10] Shimizu Y., Inoue A., Tomari Y. (2001). Cell-free translation reconstituted with purified components. Nat Biotechnol.

[11] Kurylo C.M., Alexander N., Dass R.A. (2016). Genome Sequence and Analysis of Escherichia coli MRE600, a colicinogenic, nonmotile Strain that lacks RNase I and the type I methyltransferase, EcoKI. Genome Biol Evol.

[12] Krinsky N., Kaduri M., Shainsky-Roitman J. (2016). A Simple and rapid Method for Preparing a cell-free bacterial Lysate for protein synthesis. PLoS One.

[13] Maguire B.A. (2015). Isolation of ribosomes by chromatography. Cold Spring Harb Protoc.

[14] Failmezger J., Rauter M., Nitschel R. (2017). Cell-free protein synthesis from non-growing, stressed Escherichia coli. Sci Rep.

[15] Grasemann L., Lavickova B., Elizondo-Cantu M.C. (2021). OnePot PURE cell-free system. JoVE.

[16] Ederth J., Mandava C.S., Dasgupta S. (2009). A single-step method for purification of active His-tagged ribosomes from a genetically engineered Escherichia coli. Nucleic Acids Res.

[17] (1969). M R.A Genetic locus for ribonuclease I in Escherichia coli. J Bacteriol.

[18] Beauclerk A.A., Cundliffe E., Dijk J. (1984). The binding site for ribosomal protein complex L8 within 23 s ribosomal RNA of Escherichia coli. J Biol Chem.

